# Meaningful Work, Well-Being, and Health: Enacting a Eudaimonic Vision

**DOI:** 10.3390/ijerph20166570

**Published:** 2023-08-12

**Authors:** Andrew Soren, Carol D. Ryff

**Affiliations:** 1 Eudaimonic by Design, Halifax, NS B3H 3L7, Canada; 2 Institute on Aging, MIDUS, University of Wisconsin-Madison, Madison, WI 53706, USA; cryff@wisc.edu

**Keywords:** meaningful work, decent work, eudaimonia, well-being, health, exploitation, Great Resignation, Quiet Quitting, burnout, eudaimonic design, positive deviance

## Abstract

Work is one of the most enduring and consequential life domains regarding how meaning and purpose impact health and well-being. This review first examines scientific findings from the MIDUS (Midlife in the U.S.) national longitudinal study that have linked work to well-being and health. Most have focused on adverse work or work conditions as influences on poor health, with a few recent findings investigating links to purpose and other aspects of eudaimonic well-being. Organizational scholarship is then selectively reviewed to show how meaningful work is often linked to motivation, performance, and commitment. Paradoxically, meaning can also lead to the exploitation and erosion of health and well-being when managed without regard for decent working conditions. Recent workplace phenomena known as the Great Resignation and Quiet Quitting underscore the societal consequences of work without meaning or adequate working conditions. Both the scientific and organizational literature are enriched by a vision of meaningful work rooted in Aristotle’s writings about virtue, ethics, and the realization of potential. Evidence-based practices tied to these eudaimonic ideals are examined at multiple levels, including the societal context (public policy), organizational conditions (culture, human resource practices, leadership), and individual strategies to find meaning, engagement, and fulfillment in work. A concluding section highlights strengths and omissions in the scientific and organizational literature and, going forward, calls for greater interplay among researchers, practitioners, and policymakers in enacting eudaimonic ideals.

## 1. Introduction

Work is of widespread interest in multiple domains of inquiry, and rightly so—in the words of the United Nations, work is crucial to a person’s dignity, well-being, and development as a human being [1]. This article brings two large and mostly separate bodies of work to the topic. The first pertains to scientific studies of work, well-being, and health with a focus on the MIDUS (Midlife in the U.S.) national longitudinal study, which is unique in terms of its comprehensive assessments of work experience, hedonic and eudaimonic aspects of well-being, and diverse health outcomes. While some inquiries therein have examined work influences on meaning and purpose, most findings have investigated how adverse work experiences (job insecurity, job stress, perceived unfairness) compromise mental and physical health. These adverse experiences are at odds with the call by the International Labor Organization (ILO) for decent and productive work that fosters conditions of freedom, equity, security, and dignity for all.

The second section then reviews organizational scholarship, which shows extensive engagement with the idea of meaningful work on outcomes such as worker motivation, performance, and commitment. Much of this literature posits that meaningful work leads to increased well-being, but it also considers the possibility of exploitation of those who are deeply committed to the work they do. This is especially the case when organizational culture is structured to manage meaning without establishing conditions of decent work, as defined by the ILO statement above. The importance of considering both meaningful and decent work is explored through a discussion of recent workplace phenomena known as the Great Resignation and Quiet Quitting. Both reveal that, when given the opportunity, workers may choose to find meaning in their work, even if it means doing less of it.

Both the scientific and organizational scholarship literatures benefit from embracing a broader eudaimonic vision. Thus, the third section returns to ideas articulated over 2400 years ago by Aristotle, along with historical extensions of his key messages. Of particular importance is the emphasis on ethics, virtue, and personal excellence—i.e., becoming one’s best self through personal growth and realization of potential. Given work’s significance to most lives, these dimensions underscore that work must offer both *freedom from* the kinds of adverse health and psychological risks identified in the MIDUS findings, while also offering *freedom towards* autonomy, dignity, and the development of human capabilities. Multiple levels are required to enact these ideals. At the societal level, public policy action is required. At the organizational level, culture, human resource practices, and leadership behavior must be considered. Moreover, at the individual level, strategies that promote both meaning and self-realization, while also prioritizing worker rights and needs, are necessary. Evidence-based strategies are required at each level.

The final section summarizes key themes, with emphasis on what each previous section brings forward and leaves out. When eudaimonic ideals are considered, both the scientific and organizational literature reveal opportunities where research, practice, and policy might promote the well-being and health of individuals at work for the betterment of society.

## 2. Work, Well-Being, and Health: Findings from MIDUS

This section examines how various aspects of work (defined here as paid employment) have been linked with diverse indicators of well-being and health, drawing on findings from the MIDUS (Midlife in the U.S.) national longitudinal study. The rationales for focusing on MIDUS are: (1) the study is based on a nationally representative sample, thus bringing important sociodemographic variability into research on work, well-being, and health; (2) the study is longitudinal in nature (across multiple decades), thus allowing for assessments of change in health and their antecedents; (3) the study has unprecedented richness in assessments of well-being, including purpose and meaning, as well as wide-ranging assessments of health (self-report, biomarkers); and (4) the study attends to the work-family interface, which is of increasing interest in health and organizational literature.

What follows is a brief look at illustrative findings, organized around various topics. This overview is followed by a summary that highlights ways in which MIDUS addresses questions that are similar to or different from those considered in the organizational literature, which are then covered in the next major section.

### 2.1. How Work Has Been Linked with Physical and Mental Health

Multiple MIDUS investigations have linked different aspects of work to well-being and health. Burgard, Brand, and House [2] combined data from MIDUS with data from the American Changing Lives (ACL) study to show that, at the beginning of the economic recession in 2008, persistent perceived job insecurity was a significant and substantively important predictor of poorer self-rated health and increased depressive symptoms. These results were robustly linked to controls for sociodemographic factors and job characteristics as well as earlier health and health behaviors. The findings were interpreted in the context of the industrial shift from manufacturing toward service industries and rising global competition. Other MIDUS findings showed longitudinal links between job strain (e.g., low control, high demands, low support, job insecurity) and long working hours with moderate to severe suicidal ideation [3]. Lee, Mogle, Jackson, and Buxton [4] found that perceived unfairness at work (e.g., not having work respected, not having rewarding work, perceiving that others have better jobs) was longitudinally associated with greater symptoms of insomnia over time, and that these effects were further mediated by negative work-to-family spillover. Another study [5] found that perceived job insecurity predicted poorer subjective sleep quality with the effects similarly mediated by negative work spillover.

Focused on physical health outcomes, Choi, Schnall, and Yang et al. [6], linked sedentary work with low physical demands to profiles of obesity. After controlling for multiple factors (sociodemographic factors, psychosocial working conditions, health status, health behaviors), low physical activity at work significantly predicted total and central obesity among middle-aged male workers. Combining data across 19 cohort studies from multiple countries (including MIDUS), Ferrie, Virtanen, and Jokela et al. [7] found that high job insecurity was associated with an increased risk of incident diabetes compared to those with low job insecurity, after adjusting for extensive baseline covariates. Finally, drawing on theories of work stress, Gonzales-Mulé and Cockburn [8] examined work characteristics that were linked with mortality over time. They found divergent pathways: job demands were associated with an increased likelihood of death via poor mental health when job control or cognitive ability was low; alternatively, a decreased likelihood of death was evident via better health when job control was high. We return to these ideas below when describing the relationship between dignity and meaning at work.

Work has also been examined as a social determinant of racial disparities in health. Using MIDUS data, Montgomery and Grzywacz [9] showed that three of nine job characteristics systematically varied by race: technical skills, resource management skills, and structural job characteristics (e.g., work hours, time pressure, level of competition)—were all higher in Whites. In addition, these characteristics were positively associated with eudaimonic well-being in White workers, but were negatively linked, or not associated with well-being, for Blacks. Overall, the patterns highlighted racial inequalities in eudaimonic well-being tied to the challenges Black individuals face in finding jobs that are beneficial for mental health. That is, Black individuals have fewer opportunities to acquire and hone advanced skills at work, including higher-level decision-making that is widely linked with other health outcomes (hypertension, cognitive functioning). On the positive side, advanced educational attainment was found to benefit multiple dimensions of well-being among Black workers, but showed benign or negative links for Whites, after controlling for sociodemographics.

### 2.2. Issues of Basic Needs and Directionality in Linking Work to Well-Being

Kim, Fouad, Maeda, and colleagues [10] examined three possible needs that work provides—survival (financial security), relatedness (coworker support), and self-determination (control at work, such as in decision-making). These were linked with eudaimonic well-being [11], measured as a composite. Using data from MIDUS, they found that a basic level of financial security (survival need) was more strongly linked to eudaimonic well-being overall than the need for relatedness or social determination. They discussed the practical implications of these findings for vocational psychologists and career counselors—namely, the importance of considering different needs that work provides, especially financial security, and how they are tied to diverse aspects of well-being. These themes will be reinforced below as we consider the dark sides of deeply meaningful work.

Employment may also contribute to a sense of purpose in life in that work roles can provide life direction, nurture goal-related engagement, and allow individuals to fulfill meaningful aims. Weston, Hill, and Cordador [12] investigated specific work characteristics (skill variety, autonomy, coworker support, supervisor support) as predictors of employees’ sense of purpose. Using national longitudinal data from MIDUS, they found that greater skill variety and coworker support were associated with higher levels of purpose. No linkages emerged between autonomy or supervisor support with purpose in life. A further finding was that increases in purpose over time were associated with higher *initial* levels of skill variety; that is, work experiences that from the outset allowed for and engaged different kinds of tasks and competencies. Dull, monotonous, and repetitive work thus emerged as antithetical to nurturing a sense of purpose and meaning, a point that has relevance as we consider recent workforce trends related to attrition and disengagement.

Among those who study employment experiences and psychological well-being, an important question is whether relationships between the two are bidirectional over time, or are predominantly unidirectional (employment predicts well-being, or well-being predicts work experiences). Using longitudinal data from MIDUS, Chia and Hartanto [13] examined cross-time associations between employment status and different domains of well-being, controlling for stable within-person variables (sociodemographics). Their cross-lagged panel analyses showed that employment status was not associated with various aspects of well-being at a later time point, but alternatively, that greater well-being at a prior time was associated with an increased likelihood of being employed at a later time point. The two aspects of well-being showing these effects were meaningfulness of society (the perception that life is generally sensible, understandable, and controllable) and personal growth (the sense that one is continually developing, growing, and expanding as a person over time). That higher well-being in these domains predicted subsequent likelihood of employment is a topic worthy of continued investigation on the road ahead.

### 2.3. The Work-Family Interface, Well-Being, and Health

Work is a common source of stress for many adults, arising from situations that occur at work (job demands) as well as the worries and responsibilities that people take home with them. Considerable MIDUS research has investigated the work-family interface and its connections to well-being and health, often by investigating the mediating mechanisms and moderating influences. For example, coping strategies were examined as factors that moderate linkages between work-family spillover and life satisfaction [14], and it was found that the negative association between negative spillover and life satisfaction was reduced among those who used problem-focused coping. Another study [15] linked work-home enrichment to health via the mediating role of persistence in goal striving. Individuals who reported high levels of work-home enrichment reported better health (fewer chronic conditions and health problems even when facing difficulties and lower vulnerability to stress) when they had high levels of persistence in goal striving. Another study [16] tested links between work-family conflict and four outcomes (perceived health, self-esteem, income, family support) using perceived control as a mediator. The findings showed that change in family-work conflict over 10 years predicted subsequent change in perceived control over 20 years, which, in turn, predicted lower levels of health, self-esteem, income, and family support.

In another inquiry, Tsukerman, Leger, and Charles [17] found that higher negative work-family spillover at baseline was related to more chronic illnesses, greater functional decline, and poorer self-rated health 10 and 20 years later, although job demands were unrelated to any health indices at baseline or later. Bringing in biomarkers. Versey and Tan [18] linked negative work-family spillover to metabolic syndrome (blood pressure, triglycerides, body mass index, glucose levels). Negative spillover at baseline predicted higher BMI a decade later, with marginally significant effects for triglycerides, while increases in spillover also impacted these outcomes across time. Another inquiry examined work-family enrichment as a possible buffer against inflammation among black adults [19]. Blacks, for whom cardiovascular disease is a leading cause of death, had higher concentrations of IL-6, CRP, and fibrinogen than Whites. A significant inverse association was found between work-family enrichment and inflammation as well as IL-6 concentrations, though strong variation between racial groups was not evident.

Family problems have also been linked to work-family conflict. For example, spousal problems (poor physical health, poor mental health, behavioral disorders) were linked with higher family-work conflict with relationship strain playing a primary role as mediator [20]. Women’s caregiving responsibilities (raising children, providing support to parents) were also found to influence work-family spillover in ways that varied by age [21]. Transitions related to parenting (becoming a parent, parenting children of different ages) were also found to predict both negative and positive spillover from home to work and vice versa [22]. Considering distant influences, maltreatment in childhood was found to predict work-to-family interference as well as family-to-work interference [23].

Nonstandard work schedules have effects on work-family spillover [24]—night work was specifically associated with greater marital instability, and more negative family-to-work and work-to-family spillover than daytime or weekend work. Weekend workers also reported more daily work stressors than weekday workers. Alienation in the workplace has been studied [25], focusing on levels of autonomy or creativity that work does or does not provide. For men, these qualities were linked with allostatic load, a summary index of biological dysregulation, whereas for women, alienation was linked with cognitive outcomes (memory and executive function).

Taken together, extensive findings have linked the work-family interface with diverse indicators of mental and physical health, although purpose and meaning have not been prominent in these inquiries. Some of these findings help to clarify why, when work becomes deeply meaningful, it can paradoxically become a liability for mental health and well-being, a topic to which we return in the section on organizational scholarship.

### 2.4. Entrepreneurial Pursuits and Eudaimonic Well-Being

Entrepreneurship is relevant in linking work and well-being, particularly purpose and meaning, because of its emphasis on self-initiated employment. Using data from a Portuguese sample and MIDUS, Gish, Guedes, Silva, and Patel [26] employed latent class analyses to reveal that profiles of eudaimonic well-being, personality, and temperament differed between entrepreneurs and wage earners. Underscoring relevant contextual challenges, they summarized the findings as showing that “the arc of self-employment pursuits bends toward engagement and reflexivity with deeply held values and beliefs that improve subjective experiences of eudaimonia” (p. 11). Another inquiry [27] probed the childhood adversities (neglect, abuse, poverty) among over 500 entrepreneurs in MIDUS to examine the idea that many entrepreneurs may have had difficult childhoods. The findings showed a U-shaped relationship between childhood adversity and career success that was mediated by resilience (assessed as eudaimonic well-being). Such findings underscore that aspects of well-being are relevant, not only as outcomes of entrepreneurial endeavors, but also as intervening influences between pre-work life adversity and success in self-initiated work pursuits.

A prior review of the entrepreneurial literature [28] showed that self-employment has been extensively linked to hedonic indicators of well-being (life satisfaction, contentment). Another review [29] called for greater consideration of eudaimonic well-being in formulating entrepreneurial success, which has traditionally been defined in terms of economic profit. Key questions are whether entrepreneurs view themselves as purposefully engaged in what they do, see themselves as growing and making the use of their talents and capacities over time, have a sense of being effective in managing their environment, and, importantly, view themselves as self-determined and independent. Important is the need to distinguish between opportunity versus necessity entrepreneurs, thereby illuminating differing degrees of choice in self-employment as well as differences in human capital (e.g., educational status, wealth). Where and how different aspects of eudaimonic well-being matter in the entrepreneurial journey were also considered. These ideas were elaborated by Shir and Ryff [30] through a description of the differing phases of entrepreneurship (envisioning, planning, implementation, evaluation) and what each might mean for diverse aspects of eudaimonic well-being.

Consistent with the emphasis on health in this review, Ryff [29] also called for greater focus on the health (physical and mental) of entrepreneurs—i.e., probing how high workloads and business risk might create stress, including at physiological levels. Of equal importance is how entrepreneurs impact the well-being and health of others, including those they employ as well as the communities in which they are embedded, and their own proximal family relationships. Given that eudaimonic well-being derives from central concerns with ethics, distinctions between virtuous versus malevolent entrepreneurs are also worth considering, such as through the historical literature that distinguished between productive, unproductive, and destructive entrepreneurial activities [31]. An enduring challenge is to manage the competing tensions between work that generates profit for business owners and leaders versus work that nurtures the lives and well-being of employees.

### 2.5. Summary and Transition to the Organizational Literature

This section examines prior research linking work to well-being and health in the MIDUS national longitudinal study. Findings show how various aspects of work (job insecurity, job strain, time pressures, sedentary work) are linked with diverse aspects of mental (e.g., depressive symptoms, insomnia) and physical health (e.g., obesity, hypertension, mortality). Most endeavors have not been about purpose, meaning, or other aspects of eudaimonic well-being, although such measures are available in MIDUS and could be examined. Greater skill variety and coworker support have nonetheless been linked with higher levels of purpose. Numerous investigations have focused on the work-family interface as a source of interference or enrichment, as well as how it is linked with physical health (self-reported, biomarkers) and psychological outcomes (self-esteem, cognitive function). Another line of inquiry has examined self-employment (entrepreneurship), which uniquely implicates ideas of purpose, personal growth, autonomy, and other aspects of eudaimonic well-being.

Taken together, the MIDUS findings reveal a concerning contrast with the United Nation’s call for decent work that is “productive and delivers a fair income, security in the workplace and social protection for all, better prospects for personal development and social integration, freedom for people to express their concerns, organize and participate in the decisions that affect their lives and equality of opportunity and treatment for all women and men” [32]. That is to say, large scientific studies, such as MIDUS, cover some, but not all, components of decent work.

The next section shifts toward the realm of practice via organizational studies of meaningful work. This area of the literature has not focused much on health as an outcome, thereby underscoring the relevance of the above MIDUS findings. Alternatively, the organizational literature has better formulated what meaningful work might entail and how it matters in terms of motivation, performance, and commitment. In addition, the organizational literature addresses topics largely absent in the above research—namely, the degree to which meaningful work can lead to exploitation as well as how aspects of organizational culture matter for worker experience. Post-pandemic societal changes in the work context that have led to the Great Resignation and Quiet Quitting are also covered in recent organizational scholarship, though they have received limited attention in the above science linking work to well-being and health. These differing foci underscore the need for greater dialogue between these largely separate domains of inquiry.

## 3. Meaningful Work and Its Outcomes in Organizational Scholarship

The topic of meaningful work has received increasing attention within organizational scholarship. Burghardt and Möller [33] recently reviewed publications on the topic in the management literature, finding a rapid rise in the number of studies over the past 20 years with most articles published since 2019. Over that time, excellent systematic reviews of the literature have been conducted on meaningful work [34,35,36,37,38,39,40] and the discussion that follows is largely based on the sum of those efforts. Given the focus of the journal and this special issue, we emphasized the application of theory and research in practice and on themes that related most to eudaimonic well-being and, in the absence of significant focus on health implications, that hint towards downstream health consequences (e.g., stress and burnout).

Generally, organizational scholarship has focused on positive outcomes associated with meaningful work for both individuals and organizations alike. This work has been championed in the popular press and by consulting companies seeking to help organizations capitalize on the power of purpose. However, the literature has also revealed a dark side of meaningful work—one that paradoxically seems to erode the mental health and well-being of those who find their work most meaningful. The following section reviews how meaningful work has been defined and studied within this literature, as well as what the positive and negative outcomes of meaningful work are. These considerations are then applied to contemporary workplace challenges that have been observed in the wake of the COVID-19 pandemic, with the goal of asking whether a lack of meaningful and decent work may be driving what are known as the Great Resignation and Quiet Quitting.

### 3.1. How Meaningful Work Is Defined and Studied

Studies of meaningful work in organizational scholarship have not been guided by a singular definition. In their review of the literature, Martela and Pessi [41] found 36 definitions of meaningful work and its relations, including meaning in life, meaningfulness, calling, and so on. The large overlap between meaningful work, purpose, passion, and service to some greater good has been noted [38]. Nonetheless, the most consistent approach in theory, research, and practice is Pratt and Ashforth’s [42] definition of meaningful work as work that is personally significant and worthwhile.

Much like the challenges of definition, at least 28 different measurement scales have been used to measure meaningful work [34]. Recent meta-analyses [34,43] have shown that, despite the wide variety of measures, meaningful work has moderate to large correlations with organizational outcomes, including organizational commitment [44], work and personal engagement [45], job satisfaction [44], lower intentions to quit [46], lower absenteeism [47], increased in-role [48], and extra-role [43], behaviors (e.g., organizational citizenship), employee creativity [49], and innovation [50].

Given these positive outcomes, it is not surprising that organizations have a notable interest in the potential of meaningful work to enhance motivation, performance, and commitment. Research has shown that when individuals are engaged in meaningful work, there are positive psychological outcomes, including associations with positive affect, well-being, and life satisfaction. Allan and colleagues [43] explained that this relationship through a spillover effect–positive affect is experienced when engaged in meaningful work, which improves other outcomes, and thereby enhances satisfaction with life in a virtuous upward spiral [51]. As mentioned above, eudaimonic well-being contains multiple dimensions, however, it is often essentialized as meaning and purpose by both researchers and practitioners. Only focusing on meaning becomes a way for practitioners to espouse evidence-based interventions that will boost well-being at work. Despite evidence that meaningful work has numerous positive benefits, there is also the possibility that work can have too much meaning and lead to adverse outcomes. This possibility is covered below.

### 3.2. Meaningful Work and Exploitation

Research has shown that, in certain contexts, a high degree of meaning or passion is related to the exploitation of workers and the erosion of mental health and well-being. A seminal example is Bunderson and Thompson’s [52] research on zookeepers. Most North American zookeepers view their work as a calling. Animals under their care live or die based on their efforts. In some cases, these species face extinction, creating a moral stake for the zookeepers, whose efforts are known to go over and above the call of duty. Their work is so deeply meaningful that boundaries needed to protect their well-being and mental health may be subsumed by doing whatever is needed for the care of the animals. Oelberger [53] has shown that when work is both subjectively and objectively meaningful, workers experience *boundary inhibition,* wherein “the draw of meaningfulness inhibits potential boundaries around personal space, and instead pulls their mind and body into work on an ongoing basis” (p. 571). Oelberger’s findings mirror the MIDUS research described above regarding the work-family interface and its connections to well-being and health. Boundary inhibition is one of the mediating mechanisms and moderating influences in which meaningful work can paradoxically erode well-being in certain contexts. 

The zookeepers illustrate the ‘dark side’ of meaningful work [40]. In a related study, animal shelter workers who see their work as deeply meaningful experience greater negative emotions that can result in either unfulfilling work or burnout [54]. Firefighters with a calling experience more burnout and post-traumatic stress disorder [55]. Music students with a deep sense of purpose are less likely to heed prudent career advice [56]. Those who are most loyal to their organizations are paradoxically the most exploited [57]. Those who perceive they have a calling but are unable to live it, report higher degrees of regret and lower life satisfaction [58,59]. Other professions that score high around meaning, such as health care [60,61] education [62], or non-profit, non-governmental, and charitable sectors, also demonstrate increased rates of burnout and compassion fatigue.

As Schein [63] argued, organizational culture is created through the beliefs workers have about their jobs, values that are espoused and enacted, and artifacts such as organizational practices and policies. These factors can become mutually reinforcing mechanisms that lead to either virtuous or vicious cycles. Across seven studies and a meta-analysis, Kim and colleagues [64] experimentally demonstrated this legitimization of exploitation amongst those who are perceived as highly passionate about their work. To illustrate, like many who see their work as a calling, some zookeepers declared to Bunderson and Thompson [52] that they were so passionate about what they do, they would do it for free. Hussain and colleagues [65] might suggest that those zookeepers, like many job candidates seeking social impact work, felt it was appropriate to be paid less because good work has its own intrinsic rewards. Those beliefs may reinforce those of zoo administrators who shape organizational policies that keep most zookeepers living at the poverty line [52]. A similar effect has been observed around international aid workers [53].

The above examples align with theoretical claims made by sociologists and critical theorists that active management of meaningful work can be cynically used as a means of enhancing motivation, performance, and commitment [45,66]. This has been called the *symbolic manipulation of meaning* [67], the *colonization of consciousness* [68], or *governing the soul* [69]. If workers can be convinced that their work is meaningful, their sense of self and personal identity may be manipulated, molding not only behaviors, but also feelings, aspirations, and deeply held beliefs. As Bailey et al. [70] (p. 421) describe: “under specific circumstances, the authentic and ethical intent of meaningfulness strategies can become subverted to the needs and wishes of a powerful elite, leading employees to experience alienation and dissonance between the reality they observe in their daily working lives and the rhetoric of the corporation”. These findings demonstrate that the dark side of meaningful work has the potential to erode well-being, especially when meaning is used by organizations as a justification for poor working conditions or exploitation.

### 3.3. Meaningful Work, the Great Resignation, and Quiet Quitting

To summarize, most organizational scholarship on the topic points to the positive potential of meaningful work to boost individual and organizational outcomes. When meaningful work is harnessed as a resource to be leveraged, it can also be a context for exploitation. Workers care so deeply about what they do that they endure inadequate pay, job strain, and excessive demands. The organizational literature has shown how these conditions lead to stress and eventually burnout. The MIDUS research described above further demonstrates that working conditions such as these are linked with diverse aspects of mental (e.g., depressive symptoms, insomnia) and physical illness (e.g., obesity, hypertension, mortality). All of which underscores that even if work is meaningful, if it is not decent (i.e., adhering to the ILO’s criteria), it can erode health and well-being. We argue that this progression relates to post-pandemic workforce phenomena known as the Great Resignation and Quiet Quitting, which represent widespread reactions to work that are neither meaningful nor decent.

In March 2020, lockdowns due to the COVID-19 pandemic required workers other than those deemed essential to retreat to their homes and figure out how to work from home. In the year that followed, as restrictions lifted, many employers had large numbers of jobs to fill, but struggled to bring back those who had been previously employed. The “Great Resignation”, a term attributed to Anthony Klotz [71], quickly spread across traditional and social media as a descriptor of this phenomenon. In a review of the popular and research literature on the topic, Miller and Jhamb [72] noted that the Great Resignation was most significant among in-person, low-wage, and younger workers. Industries with higher wages and a greater propensity to support remote workers were largely spared [73]. Empirical data from the U.S. showed that pandemic-related implications on the job market have been markedly different from other macro-economic events such as the dot-com recession of 2000–2001 and the Great Recession of 2007–2009 [74]. These comparisons suggested that the Great Resignation may have been more of a great reshuffle—i.e., a case of supply and demand that gave workers facing indecent work new opportunities to find better working conditions in response to burnout, unfair workplace mandates, inadequate compensation, and/or toxic workplace cultures [75].

Even when workers did not choose to resign, by the second quarter of 2022, Gallup reported that over 50% of U.S. employees were disengaged and disillusioned [76]. This phenomenon became known as *Quiet Quitting:* opting out of tasks beyond one’s assigned duties and/or becoming less psychologically invested in work. Quiet Quitting is not novel. Similar job action has been called *instrumental compliance* [77], *resigned behavior compliance* [78], or simply work-to-rule. In a survey of 1200 HR professionals from the Society for Human Resource Management mid-way through 2022, 51% indicated that Quiet Quitting was a concern, 36% said it was actively occurring in their organizations, particularly among younger and hourly wage workers, and 60% said that the problem was post-pandemic culture [79].

As a widespread phenomenon, Quiet Quitting is a risk for organizations that rely on workers’ discretionary effort to go over and above the call of duty [80]. Such citizenship behavior typically benefits employees as well as their organizations [81]. Employees often experience greater social connection, better career growth, and higher well-being when they engage in citizenship behaviors. The organization benefits, in turn, through increased productivity, innovation, and service, ultimately lead to competitive advantage [82]. When employees disengage and do the minimum required, those benefits go away.

It has been noted that the Great Resignation and Quiet Quitting reflect broader feelings of interpersonal injustice at work [83]. While many organizations made great efforts to say they cared about the well-being of workers over the course of the pandemic, most people can readily identify an incongruence between espoused and enacted values at work [66]. To create conditions that foster decent, meaningful, and self-fulfilling work, organizations must put values into action through concrete organizational policies and practices, which uphold those values and hold leaders accountable for demonstrating them. Reinforcing the point, a study from over 34 million online employee profiles [84] found that toxic corporate culture, characterized by a disregard for diversity, equity and inclusion, disrespect, and unethical behavior was 10.4 times more likely to contribute to attrition during the Great Resignation than compensation.

In the face of job insecurity or organizational politics, frustrated workers might not resign even if they are morally outraged by the incongruence they observe [85,86]. Thus, Quiet Quitting, as Detert [87] observed, could be a means of “calibrated contribution”—i.e., an act of self-determination in the context of multiple grim realities. These include: (1) minimum wage in the U.S. (adjusted for purchasing power) being at its lowest point in 66 years; (2) the average ratio between CEO and median worker pay was 670 to 1 in 2021; (3) income inequality in the U.S. currently reflects levels traditionally seen in economically undeveloped or extremely corrupt countries; (4) unions working for better pay and working conditions are at their lowest participation since 1955, and (5) no significant federal legislations to strengthen worker rights is in sight [87].

### 3.4. Summary and Transition to a Eudaimonic Vision

The organizational literature reveals diverse formulations of meaningful work that have been examined as possible influences on worker motivation, performance, and commitment. Unfortunately, there is a dark side to meaningful work, especially when certain beliefs, values, and policies that legitimize the exploitation of meaning become woven into organizational culture and despoil decent work. Such topics are largely missing in scientific studies of work, well-being, and health—even though they demonstrate that unfair job demands, inadequate compensation, and toxic corporate culture can lead to worker dissatisfaction and eventually burnout. Understanding the relationships between how work is designed and what impact that has on peoples’ health and well-being is critical. The Great Resignation and Quiet Quitting, recent workforce occurrences that emerged from the pandemic, suggest that the status quo has detrimental societal and economic consequences when work lacks both meaning and decency. After all, when given the opportunity, workers will use whatever power they do have to make work meaningful, even if it means doing less of it. We are still waiting for scientific research on work, well-being, and health to explore these more recent historic events.

Despite the depth and richness of the scientific and organizational literature, neither has explored the full potential of eudaimonia at work. Eudaimonic well-being is characterized by multiple dimensions including personal growth, autonomy, environmental mastery, positive relationships, self-acceptance, and purpose in life, but too often is distilled by both researchers and practitioners as just meaning at work. The consequence is that many rich ideas woven into ancient scholars’ conceptions of eudaimonia are missing. The next section revisits these ideals and their subsequent elaborations. The relevance of this broader vision for promoting work that is dignified, meaningful, and self-fulfilling is then considered. Included is a call for virtue and ethics to be applied at multiple levels, including societal preconditions that require beneficent public policies as well as eudaimonic-inspired organizational practices and personal strategies. We examine intentional examples of such practices that depart from the norm in honorable ways, what Spreitzer and Sonenshein [88] defined as positive deviance in positive organizational scholarship, thus pointing to promising future directions. The over-arching objective is to elevate the discourse on work, well-being, and health by embracing core ideas of eudaimonia.

## 4. Bringing a Larger Eudaimonic Vision to the Experience of Work

### 4.1. A Return to Eudaimonic Ideals and Their Historical Extensions

Aristotle’s notion of eudaimonia referred to the potentiation of that good spirit or daimon within us all that is called to purpose through the activity of the soul in accordance with virtue [89]. This classical conception of a calling suggests that each person is destined to fulfill a certain purpose; hence, one has a duty to find and embrace one’s destiny and work to potentiate it. Such thinking was embraced by early Christianity, which replaced the potentiation of individualized purpose with the singular calling of God towards service in the name of the church [40]. That changed with the Protestant Reformation of the sixteenth century when Martin Luther asserted that secular work—not just the work of the ministry—constituted a calling in life. This proclamation brought meaningful work back to the masses by maintaining the core elements of destiny, duty, and discovery, without needing the divine caller [90]. It must be admitted that this eudaimonic frame remains narrowly located and is only a starting point for incorporating other voices and experiences that transcend this euro-centric reading. Nonetheless, from the Enlightenment onward, the idea that work can and should be meaningful has increasingly flourished into the protestant work ethic so prevalent across much of the globe today.

In the twentieth century, and especially following the Second World War, theory and practice around meaningful work grew in popularity as existentialists and humanists returned to Aristotelian foundations of potentiation and self-actualization. Frankl [91] articulated the life-sustaining value of purposeful life engagement and proposed that every person has a will to meaning, thus framing meaning as an intrinsic part of being human. Jahoda [92] applied the idea to work, suggesting that workers are compensated not only with financial benefits, but also with the satisfaction of basic psychological needs such as social relatedness and purpose, both of which are fundamental to our well-being. Herzberg [93] popularized this notion formulated as a two-factor motivational model that determined either satisfaction or its opposite at work, largely based on the degree to which jobs fulfill intrinsic needs such as personal growth, passion, social responsibility, opportunity for growth, respect, recognition, and achievement. These ideas were also central to Karasek’s [94] job demands-control model, a widely studied model of occupational stress. Key to this model, implicated in some of the MIDUS research, is the belief that the strain an employee feels at work due to job demands is buffered by their capacity to control the situation—thus placing an emphasis on environmental mastery and personal growth.

Later, these ideas were integrated with conceptions of human becoming from developmental, existential, and humanistic psychology in a model of psychological well-being [11] that encompassed qualities such as purpose, personal growth, autonomy, and mastery. That is to say, Aristotle’s eudaimonia nourished thinking about what constitutes positive psychological functioning as well as what defines meaningful work. Both are of central concern in this review.

It is important to underscore that Aristotle was singularly concerned with virtue and ethics. A central question is how to bring these critical issues to the realm of meaningful work today. One way is to consider this progression: if potentiation of the self is the ultimate eudaimonic goal of life, and if work is a primary vehicle towards achieving this goal, then the potential of work for emancipation or alienation of meaning, purpose, and self-realization is of moral, ethical, and political relevance [36,95]. Political theorists have argued that, for work to be meaningful, it must offer *freedom from* arbitrary interference or domination, specifically fear of physical and psychological harm [96]. These are the very work demands underscored in the MIDUS research described above that led to negative health and well-being outcomes. However, work should also offer *freedom towards* autonomy and dignity [37]; that is, provide workers with the freedom to take personal responsibility to choose right over wrong [97] and to self-realize by developing their capacities in pursuit of valuable purposes. This form of *freedom to* becomes a rallying cry from those such as Marx [98], who considered a just society one wherein work empowered individuals to develop and apply their innate skills, capabilities, and desires within a community striving for the betterment of all; to Nussbaum [99], whose capabilities approach to human development is embraced by the United Nations today as a necessary precondition to fostering dignity at work and in life more broadly.

This eudaimonic reading illuminates why framing meaningful work as personally significant and worthwhile [42] fails to address the necessary complexity of work’s role in our lives. It only attends to the individual, without taking the broader system in which they exist into account. That systems lens is essential for ethical research and application [100,101,102]. Researchers and practitioners must move from a focus on just *meaning* at work, to that of *just* meaning at work. The most effective framework that corrects this over-simplification is Duffy and colleagues’ Psychology of Work Theory [103], which posits a critical inter-relationship between meaning and decency as applied to multiple levels of an overarching system. In other words, this eudaimonic lens should be applied as a societal precondition (implicating public policy), organizational conditions (including culture, practices, and leadership) as well as individual strategies that might be designed to nurture work that has dignity, meaning, and contributes to self-realization. While this formulation may feel complex and abstract, there are tactical and practical strategies from the world of organizational scholarship that illustrate the kinds of evidence-based practices that might be considered to bring this eudaimonic vision to life.

### 4.2. The Societal Preconditions of Meaningful Work

The United Nations’ International Labor Organization typifies the eudaimonic framework when it describes work as “crucial to a person’s dignity, well-being, and development as a human being” [1]. This aspiration is far from reality across much of the globe [103,104] and, importantly, is not just a developing world problem. There are a plethora of *lousy jobs* [105], or *bullshit jobs* [106], failing to meet the ILO’s standards that fuel supposedly first-world economies where both meaning and dignity are absent. It is important to consider that these may be the conditions that fed the Great Resignation and Quiet Quitting described above.

While it is possible to have one without the other, meaningful work and decent work often interact [35]. Decent work is best viewed as a societal antecedent of meaningful work. When shareholders demand ever-greater quarterly stock returns, or voting citizens demand public actors to be more efficient with fewer tax dollars, however, organizations are put in situations where meaningful work becomes an efficiency strategy for extracting more discretionary effort from the workforce at less expense. It is thus at the societal level that there is a great need for responsible policy and regulatory interventions to uphold eudaimonic intentions and moderate the potential of exploitation sometimes associated with meaningful work.

Recently, in the United States, the Surgeon General released a Framework for Mental Health and Well-Being in the Workplace [107] that represents true positive deviance at the societal level. The set of recommendations centers on the worker’s voice and equity, outlining the foundational role that workplaces should play in promoting the health and well-being of those they employ. Five pillars are included in the framework: (1) *Mattering at Work*, focuses explicitly on the intersection between dignity and meaning and calls on organizations to provide a living wage, engage workers in workplace decisions, build a culture of gratitude and recognition, and connect individual work with the organizational mission; (2) *Opportunity for Growth*, which underscores training, education, and mentoring, while fostering pathways for career advancement, and ensuring relevant and reciprocal feedback; (3) *Work-Life Harmony*, which calls for more autonomy in how work is done, including flexible schedules, access to paid leave, and respect for boundaries between work and non-work; (4) *Connection and Community*, which creates cultures of inclusion and belonging, cultivates trusted relationships, and fosters teamwork; and (5) *Protection from Harm*, which prioritizes workplace safety, adequate rest, supports mental health, and promotes diversity and inclusion.

The Surgeon General’s framework is worthy of widespread applause, converging on multiple themes emphasized in this review. That said, whether the framework will be consequential in the context of the market-driven, neo-liberal realities that most organizations compete within is of central concern. As Blustein, Lysova, and Duffy [35] argue, further government action is likely required to lower the proportion of individuals experiencing economic constraints and marginalization. Doing so will require multiple levels of change/action: providing greater and equal access to high-quality education and resources, developing laws, regulations, and policies that minimize the risk of discrimination or mistreatment and fostering inclusion within the workplace. That said, change can also come from within organizations, via the kinds of cultures, policies, and leadership expectations they endorse, as covered in the following section.

### 4.3. Organizational Conditions for Dignified, Meaningful, and Self-Fulfilling Work

At the organizational level, the design of eudaimonic work occurs in three ways: first, in the declaration of values that an organization and its members espouse; second, in the policies and practices designed to enact those values; and third, in what is done to endorse leadership behaviors likely to prioritize meaning while embracing the broader scope of eudaimonia.

Values-driven cultures that espouse *and enact* values such as care, connection, mutuality, fairness, integrity, and excellence have been linked with meaningful work [70,108]. Similarly, organizational cultures that champion collective decision-making and autonomy—where values are co-created by those who need to live them—have great potential for meaningful work [66,95,109]. With these values acting as a social contract, relationships with others often shape meaning and build belonging, social identity, and shared purpose [39,45,66]. Behaviors such as respectful engagement, support, cooperation, helping, mentoring, and trust are prized [110], as are climates of psychological safety that allow for the acknowledgment of challenge and struggle [45,111].

Even before employees are hired, meaning can be designed into jobs by being intentional about the type, quality, and amount of work to be done [112,113]. This includes factoring work-life balance and fair pay into the employment contract as enabling conditions for decent work [114]. From there, eudaimonic aims can be considered top-down efforts, such as the person-job fit [115], where managers identify job seekers with a greater potential to find their work meaningful; or bottom-up efforts, such as job crafting, where workers are given autonomy to align daily tasks to individual strengths, passions, and values [116].

Reward and recognition systems that reinforce eudaimonic behaviors consistent with organizational values are critical ways in which culture is enacted through policy [70]. The same can be said for policies and practices that support worker autonomy—whether in how goal setting and evaluation are managed [33], or in ways that daily tasks and progress are tracked. In both cases, micro-management and surveillance can be at odds with a eudaimonic intention.

Meaningfulness can also be designed into working conditions at the end of employment. Bright, Cameron, and Caza [117] studied how organizations downsize in ways that display moral excellence and ethical character. For example, when leaders take responsibility for layoffs and involve employees in the decision-making process, provide outplacement assistance, and offer retraining opportunities, they amplify acts of responsibility throughout the organization, creating a virtuous cycle associated with greater positive emotion, social capital, and prosocial behavior [118].

Leaders often bridge working conditions and meaningfulness because they play a key role in the design of work and how it is lived day-to-day, make sense of organizational culture, and help workers connect daily tasks to a greater purpose [119]. Leaders model eudaimonia when they emphasize ethical values and congruently behave with those values, thereby linking work, organizational ethical goals and standards, and broader societal outcomes [120]. In a review of leadership behaviors connected with meaningful work, Steger [121] summed up the behaviors required with the acronym CARMA. **C**larity is about architecting and communicating a meaningful mission or purpose that resonates with employees. **A**uthenticity requires being true to oneself and one’s values as a leader, while behaving ethically, honestly, transparently, and encouraging the same from others. **R**espect involves facilitating a sense of community and connectedness among employees. **M**attering is about recognizing contributions and helping individuals feel valued and valuable. **A**utonomy represents the ways in which a leader can support self-direction, trial and error, and innovation in the workplace.

In summary, positively deviant organizational conditions can be designed to foster dignified, meaningful, and self-fulfilling work in the spirit of eudaimonia. These conditions are evident in organizational cultures that espouse sustainable, human-centric values, where concrete organizational policies and practices uphold those values, and where leaders are held accountable for demonstrating them through their behavior. Creating these conditions is not easy nor simple, but doing so sets the stage for individuals to take their own initiatives to cultivate meaningful work and pursue their eudaimonic potential.

### 4.4. Individual Strategies for Meaningful Work

Lysova and colleagues [38] describe three factors that influence individual strategies for meaningful work. First are *dispositional signatures*, which refer to the individual’s interests, abilities, and traits. These represent personal resources for meaningful work that require self-awareness. Those who know themselves have a higher chance of achieving a person-job fit. In eudaimonic terms, dispositional signatures may be closest to Aristotle’s daimon or good spirit that one is always striving to potentiate. Second are *characteristic adaptations*, which refer to the goals one sets and how one is motivated to achieve them. This factor offers a great opportunity to find congruence between what is espoused and what is enacted—or what Aristotle might have described as the virtuous activity of the soul, moderated by reason. Third are *personal narratives*, which are the stories told about oneself, one’s job, and one’s organization. This factor points to people’s need to make sense of their work [122], which Foster [123] describes as *emplotment.*

Even when structurally challenging, individuals can still find meaning at work. Studies have shown that those employed in jobs scoring lower on decency, such as precarious workers (e.g., digital gig economy [124], or those who participate in dirty work (e.g., sanitary workers [125], can still create conditions for themselves to experience meaningful work. Ashforth and Kreiner [126] have shown that this is often achieved through narrative reframing (i.e., ascribing meaning to work that may otherwise be unpleasant), recalibrating (i.e., adjusting expectations or standards), and refocusing (i.e., shifting attention to non-tainted parts of the job). Indeed, some have suggested that meaning is often built in terrible situations with others [36,127], where working together in the face of bad working conditions can lead to perceptions of meaningfulness. These studies convey ways to make work satisfying and meaningful beyond managerial reach [128] because meaningful work is about human agency, or as Rosso and colleagues describe, our eudaimonic drive “to separate, assert, expand, master, and create” [39] (p. 114).

To summarize, this section began with a distillation of Aristotle’s eudaimonic vision, which has rich potential to infuse work experience with new ideals and objectives. How these might be enacted has been considered at multiple levels—societal, organizational, and individual. We highlight these as tactical and practical strategies going forward. For researchers, they point to new questions for empirical inquiry; for practitioners and policymakers, they offer new directions to build a eudaimonic-inspired workplace and a society with greater cumulative health and well-being.

## 5. Future Directions

This review has covered extensive territory. As a way of summarizing what has been presented, the final section below targets not the advances in the preceding literature on research and practice, which have already been covered, but rather the omissions and lacunae that must be acknowledged to move these realms forward. Stated otherwise, the juxtaposition of the two opening sections above—namely, illustrative scientific advances linking work to well-being and health, and the organizational scholarship focused on differing conceptions of meaningful work and the possibilities for worker exploitation—are particularly helpful in identifying what each realm of inquiry examines and neglects. The latter we showcase in this final section as a way of highlighting needed future directions. If, as the ILO suggests, work is crucial to a person’s dignity, well-being, and development as a human being, these recommendations have broader societal significance. They should inspire researchers, practitioners, and policymakers to question the principles that guide their realms of inquiry, which involve more than focusing only on measures, samples, designs, and interventions.

### 5.1. What the Science of Work, Well-Being, and Health Needs

The organizational literature reveals interesting omissions in extant research on work, well-being, and health. Because scientific studies focus on individual assessments of work (e.g., job demands, job insecurity), psychological experience (e.g., life satisfaction, autonomy, purpose), or health (e.g., chronic conditions, inflammatory markers, sleep), issues related to the larger organizational culture are neglected. This means that little is known about how workers perceive the actions of those in leadership roles—are they seen as constructive and supportive in their decisions and actions? Do workers perceive that they are respected, valued, and properly remunerated by their employers? Or do they perceive they are of peripheral concern amidst the prioritization of company profits? Stated otherwise, toxic corporate culture is a critical feature of the workplace experience, but its presence in scientific studies is limited by the fact that existing inquiries do not assess workers’ perceptions of the work culture that they inhabit. Standard assessments of work stress also give limited attention to whether workers perceive they are experiencing growth and development on the job, and whether their talents and capacities are being nurtured. The latter issues are central features of eudaimonia.

Relatedly, the five pillars of the Surgeon General’s framework on Workplace Mental Health and Well-Being warrant increased attention in extant scientific studies, particularly work-family harmony, mattering at work, and opportunities for growth. Findings from MIDUS show that the work-family interface can positively play out as enrichment between the two realms, or negatively as adverse spillover from one realm to the other, with additional links to different aspects of mental and physical health. Alternatively, opportunities for growth, as noted above, have received limited attention in the science of work context, as has mattering (dignity, meaning, recognition, adequate compensation).

Critically important in the organizational literature has been the topic of exploitation in the workplace, likely tied to the decision of many to exit the workplace during the pandemic (Great Resignation) or to do less and less on the job (Quiet Quitting). These adverse experiences are not covered by usual assessments of job demands and job stress. As such, they constitute a much-needed future emphasis on the dark side of meaningful work.

### 5.2. What the World of Organizational Practice Needs

It is not surprising that widespread interest in meaningful work has been pursued as a window on what influences work motivation, performance, and commitment. Organizational practitioners are keen to offer interventions that boost these outcomes, and if they can say that those interventions also benefit the well-being of employees along the way, so much the better. However, too often missing from the world of practice are questions about the unintended consequences of these so-called “positive interventions”. Speaking to the need for greater collaboration between research and practice, these kinds of questions are routinely included in scientific studies of work, well-being, and health. In the context of meaningful work, such questions could include asking whether workers see themselves as having greater job security or not, whether they find their jobs more stressful or less, whether their work environments support the kinds of autonomy they need to enact their personal values, or whether their work instead fosters alienation through a lack of say in how it gets done. These questions may be asked in other organizational contexts, but are rarely connected to questions of meaningful work.

It is notable that the organizational literature on meaningful work has paid limited attention to worker health. Although there is increasing attention being paid to mental *illness* in the workplace (depression, anxiety), far less attention is given to mental *health* and well-being. When well-being is a focus, it is largely considered based on simple measures of happiness or subjective well-being (positive emotion, life satisfaction) rather than more nuanced aspects of eudaimonic well-being (personal growth, autonomy, environmental mastery, positive relationships, self-acceptance, purpose in life). Physical health, both subjectively and objectively measured (chronic conditions, physiological regulation) has been notably missing in organizational studies on meaningful work. This omission is surprising, given that worker illness and absenteeism, to say nothing of worker healthcare expenses, are not in the best interests of company success and profitability.

The Surgeon General’s pillars of workplace health and well-being also need greater attention in organizational practice. Although prior emphasis has been given to safety in the workplace (Protection from Harm) and examples can be found of how some organizations emphasize each of the five pillars of the framework, the vast majority of companies would be challenged in prioritizing these pillars: to prioritize Mattering at Work, Work-Life Harmony, Opportunity for Growth, and Connection and Community.

### 5.3. What Eudaimonic Ideals Call Forth

Both science and practice are enriched by embracing the ethical and virtuous foundations of eudaimonia. If work can be seen as a vehicle to enact the unique talents, strengths, and capacities of individuals in service of meaningful goals, not only would the workplace be enhanced, but more importantly, societies would have increased levels of freedom, well-being, and human capacity. This stance matters for scientific studies of work, well-being, and health, as well as for organizational practices seeking to promote meaningful work that likely enhances worker performance. The upshot is that both realms cannot carry out the eudaimonic vision without attending to ethics and virtue.

Such ethical considerations must be pursued at multiple levels. Arguably, the societal context is most important, but it rarely gets attention. Public policy defines how societal values and priorities are enacted, but this realm is largely missing in scientific studies, which put forth numerous findings but rarely consider their relevance for extant or needed government-initiated intervention. Organizational scholarship may provide good guidance for what companies should be doing, but without public policy and regulation to provide oversight on such things as the distribution of profits to workers versus shareholders, it is unlikely that most will take the recommendations.

Organizational conditions within the workplace also matter, and these need to be examined with regard to guiding principles and practices. Do they show a commitment to nurturing work that is dignified, meaningful, and self-realizing? Or are they guided by values that foster and exemplify corporate self-interest at the expense of workers?

Similarly, individual strategies for promoting eudaimonia in the workplace that have been explored in the organizational practice literature are promising and worthy of greater adoption. Those interventions, however, should be wisely chosen and with an understanding that exclusively focusing on meaning can lead to exploitation without the right enabling conditions that foster *just* meaning at work.

## 6. Conclusions

On the road ahead, we call for research, practice, and policy that embraces a broad eudaimonic vision of work. Although extensive science has investigated how employment contributes to, or undermines, well-being and health, most of this literature has focused on adverse work conditions (job insecurity, job stress, high demands) and their consequences for poor health (mental and physical). When positive outcomes have been considered, they have often focused on hedonic aspects of well-being (positive affect and life satisfaction) or limited aspects of eudaimonic well-being (meaning and purpose in life). The net effect is that little scientific work to date has explicitly investigated the degree to which different kinds of work nurture, or undermine, individuals’ perceptions of whether they are making good use of their talents and capacities over time (personal growth), living by the own convictions (autonomy), or achieving a person-environment fit (environmental mastery) in the workplace.

Access to meaningful work and the means of cultivating it invoke notable ethical challenges in need of serious attention by researchers and practitioners. Importantly, issues of ethics and virtue have received minimal attention in how meaningful work is studied. A key principle, in the words of Isaac Prilleltensky [102], is that *there is no wellness without fairness*. That is to say, the psychosocial and organizational benefits that come from engaging in meaningful, purposeful, and self-realizing work should not be examined without also attending to issues of alienation, exploitation, and burnout. Better measures and evaluation strategies are needed to spotlight the moral conditions that enable meaningful work in a eudaimonic context.

A worthy goal going forward is that of greater collaboration between science and practice built around this eudaimonic vision. As illustrated in these pages, there are positively deviant applications of meaningful and decent work that reflect virtuous organizational practices. These practices spark meaning, while also buffering and bolstering well-being. There are valuable recommendations coming from the government that can help, such as the Surgeon General’s framework; however, regulatory pressure needs to be applied to ensure the adoption of those principles. Key questions are what can be learned from these practices and recommendations that enrich future research on what dimensions of work should be studied and how they matter for well-being and health. Scientific studies of how organizations create and sustain freedom, meaning, and the self-realization of their employees are key imperatives. Such inquiries will help practitioners working at societal, organizational, and individual levels to design policies and interventions that nourish eudaimonic well-being through meaningful work. The health benefits thereof are likely to be substantial, but quality research is needed to document for whom and under what conditions such effects occur.

## Data Availability

MIDUS data are publicly available. Information about accessing the data can be found here: www.midus.wisc.edu (accessed on 27 July 2023).
